# Intranasal Delivery of Cationic PLGA Nano/Microparticles- Loaded FMDV DNA Vaccine Encoding IL-6 Elicited Protective Immunity against FMDV Challenge

**DOI:** 10.1371/journal.pone.0027605

**Published:** 2011-11-15

**Authors:** Gang Wang, Li Pan, Yongguang Zhang, Yonglu Wang, Zhongwang Zhang, Jianliang Lü, Peng Zhou, Yuzhen Fang, Shoutian Jiang

**Affiliations:** State Key Laboratory of Veterinary Etiological Biology/National Foot and Mouth Disease Reference Laboratory/Key Laboratory of Animal Virology of Ministry of Agriculture, Lanzhou Veterinary Research Institute, Chinese Academy of Agricultural Sciences, Lanzhou, Gansu, China; College of Medicine, Hallym University, Republic of Korea

## Abstract

Mucosal vaccination has been demonstrated to be an effective means of eliciting protective immunity against aerosol infections of foot and mouth disease virus (FMDV) and various approaches have been used to improve mucosal response to this pathogen. In this study, cationic PLGA (poly(lactide-co-glycolide)) nano/microparticles were used as an intranasal delivery vehicle as a means administering FMDV DNA vaccine encoding the FMDV capsid protein and the bovine IL-6 gene as a means of enhancing mucosal and systemic immune responses in animals. Three eukaryotic expression plasmids with or without bovine IL-6 gene (pc-P12A3C, pc-IL2AP12A3C and pc-P12AIL3C) were generated. The two latter plasmids were designed with the IL-6 gene located either before or between the P12A and 3C genes, respectively, as a means of determining if the location of the IL-6 gene affected capsid assembly and the subsequent immune response. Guinea pigs and rats were intranasally vaccinated with the respective chitosan-coated PLGA nano/microparticles-loaded FMDV DNA vaccine formulations. Animals immunized with pc-P12AIL3C (followed by animals vaccinated with pc-P12A3C and pc-IL2AP12A3C) developed the highest levels of antigen-specific serum IgG and IgA antibody responses and the highest levels of sIgA (secretory IgA) present in mucosal tissues. However, the highest levels of neutralizing antibodies were generated in pc-IL2AP12A3C-immunized animals (followed by pc-P12AIL3C- and then in pc-P12A3C-immunized animals). pc-IL2AP12A3C-immunized animals also developed stronger cell mediated immune responses (followed by pc-P12AIL3C- and pc-P12A3C-immunized animals) as evidenced by antigen-specific T-cell proliferation and expression levels of IFN-γ by both CD4+ and CD8+ splenic T cells. The percentage of animals protected against FMDV challenge following immunizations with pc-IL2AP12A3C, pc-P12AIL3C or pc-P12A3C were 3/5, 1/5 and 0/5, respectively. These data suggested that intranasal delivery of cationic PLGA nano/microparticles loaded with various FMDV DNA vaccine formulations encoding IL-6 as a molecular adjuvant enhanced protective immunity against FMDV, particularly pc-IL2AP12A3C with IL-6 gene located before P12A3C gene.

## Introduction

Foot and mouth disease virus (FMDV) infections following exposure to contaminated aerosols can be prevented by neutralizing mucosal immune responses directed against FMDV antigens, suggesting that vaccines designed to elicit mucosal FMDV-specific immunity at major mucosal surfaces can interfere with viral transmission [1]. Since protection against mucosal infection has been attributed to the production of anti-FMDV-specific IgA antibodies [2], elicitation of IgA at these surfaces has been deemed an important parameter in the development of vaccines designed to elicit protective immune responses against FMDV [3].

Interleukin-6(IL-6) is a multifunctional Th2-associated cytokine produced by macrophages, dendritic cells, T cells, endothelial cells and hepatocytes [4] that plays a role in the terminal differentiation of B cells, proliferation of lymphocytes and endothelial cells, regulation of IL-2 receptor expression, differentiation of CTL responses, up-regulation of acute phase proteins, Th2 differentiation (via the upregulation of IL-4 by precursor T helper cells) and regulation of Th1-associated cytokines [5].

Since DNA plasmid vaccines used to stimulate mucosal immunity can be easily degraded by DNases present at mucosal surfaces, DNA plasmids were adsorbed onto chitosan-coated PLGA particles that were shown to be protected against enzymatic degradation [6]. For biodegradable and biocompatible characteristics, poly(D,L-lactide-co-glycolide) (PLGA) nanoparticles have been extensively utilized in the sustained and targeted delivery of various agents, including anticancer drugs [7], plasmid DNA [8], proteins or peptides [9], [10] and low-molecular-weight compounds [11]. PLGA nanoparticles have hence been used to increase the concentrations of drugs crossing various biological barriers, including the blood-brain barrier, gastrointestinal and mucosal surfaces and ocular tissues [12]. Because of its cationic nature, chitosan has been widely tested as a non-viral gene delivery system [13]. Its mucoadhesive properties and its ability to modulate tight junction integrity resulting in increased paracellular transport, make it an ideal candidate for the delivery of DNA vaccines to mucosal tissues [14]. Furthermore, chitosan-coated PLGA nanoparticles were found to increase the penetration of the encapsulated macromolecules at mucosal surfaces [12], [15].

In this study, using chitosan-coated PLGA nanoparticles as a delivery vehicle, we sought to explore whether plasmids encoding FMDV capsid protein and bovine IL-6 as mucosal adjuvant, and the different position of IL-6 among the plasmids can improve the stimulation of mucosal and systemic immune responses.

## Results

### Construction and plasmid characterization

**Figure 1:**
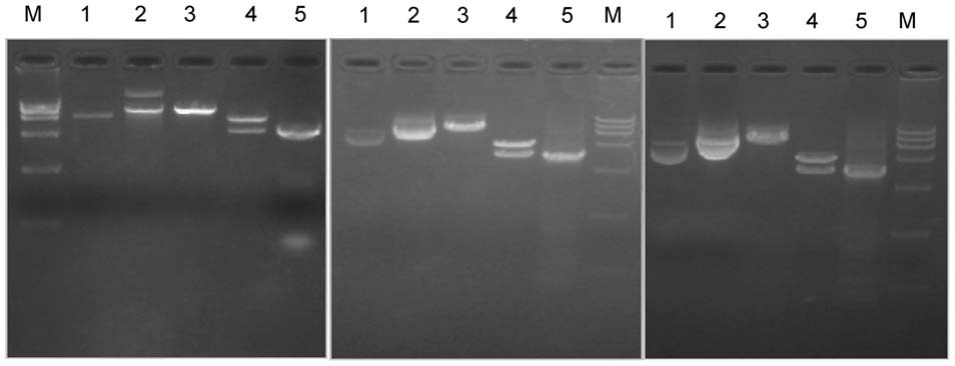
Restriction pattern profiles of digested plasmids. Plasmid pA (gel on left), pB (middle gel) and pC (gel on right) were enzymatically digested. Digests were subjected to agarose gel electrophoresis. M, DNA ladder; Lane 1, pcDNA3.1; Lane 2, undigested plasmid; Lane 3, *Eco*Rl digested plasmid; Lane 4, *Bam*HI and *Xba*I digested plasmid; Lane 5, PCR product of target gene.

**Figure 2:**
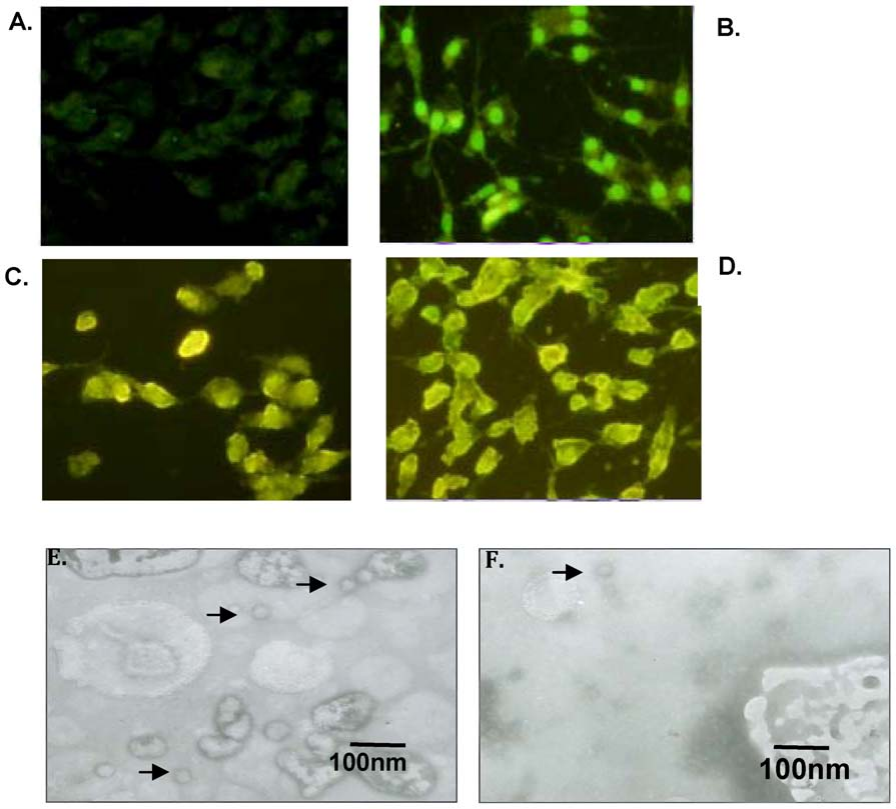
Detection of the FMDV structure protein and FMDV capsid. Immunofluoresence was used to determine the expression levels of the FMDV structure protein following transfection with either pcDNA3.1(+) (a), pA (b) pB (c) or pC (d). FMDV capsid was observed by TEM of BHK-21 cells transfected with pA (e) or pC (d).

Three plasmids were constructed successfully and confirmed by PCR, enzyme digestion (Figure 1) and sequence analysis. Plasmid expression was confirmed using an indirect immunofluorescence assay (Figure 2). Transfected cells were incubated with anti-FMDV positive sera followed by an incubation with a fluorescein-conjugated anti-rabbit IgG. As anticipated, cells transfected with pA, pB or pC fluoresced compared to the negative controls (Figure 2).

**Figure 3:**
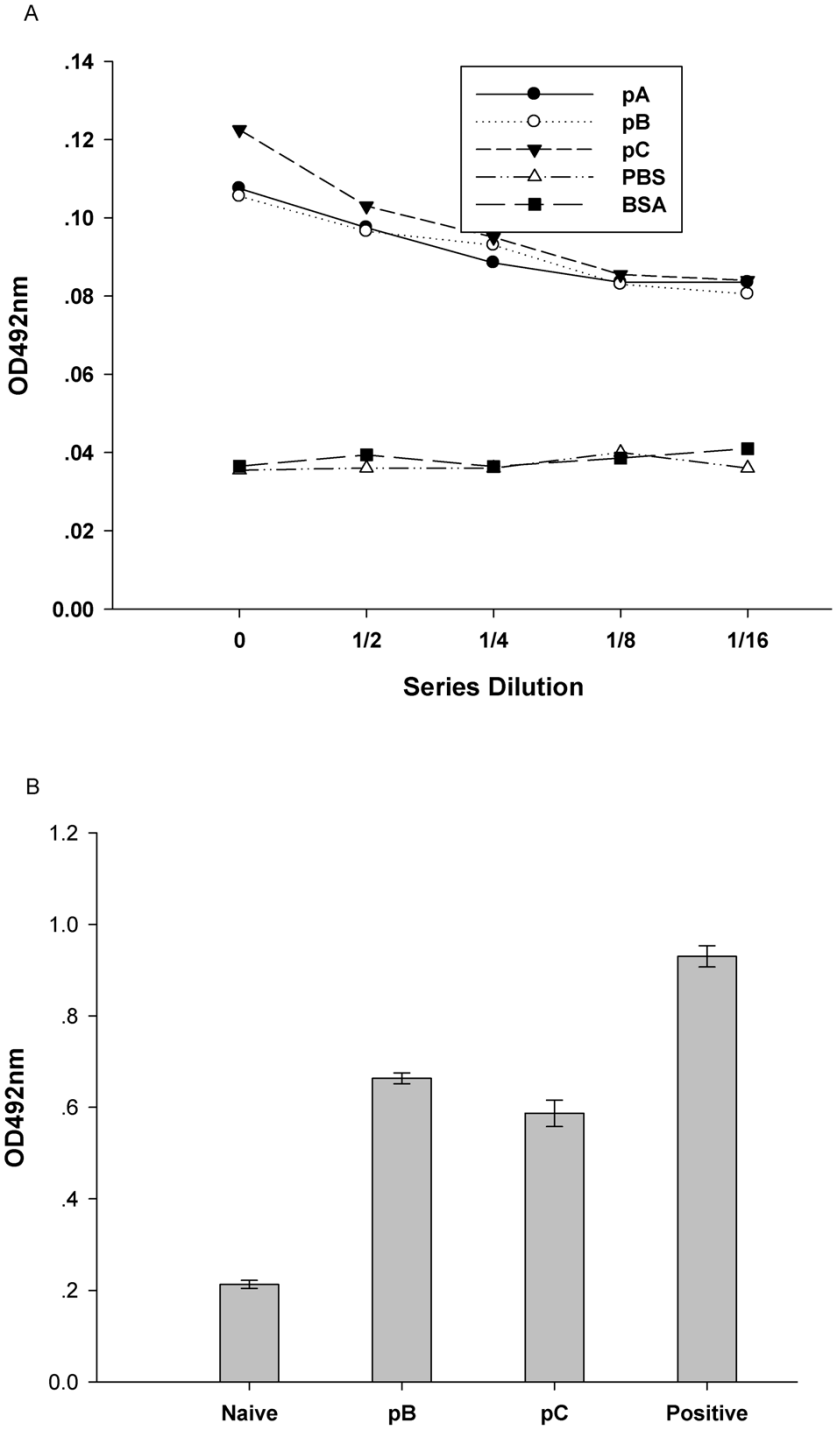
Characterization of FMDV protein and IL-6 production in BHK-21 cells. (A)Two days after transfection with either pA, pB or pC, and negative control (PBS) and irrelevant control (Bovine Serum Albumin, BSA) were added; BHK-21 cells were analyzed for expression of FMDV proteins by sandwich-ELISA. BHK-21 cell lysates were diluted twofold. The data are expressed as the mean OD for each dilution. (B) Expression of IL-6 was determined by assessing IL-6 levels in BHK-21 cell lysates by ELISA two-days after transfection. The data are expressed as the mean OD ± SEM, measured in duplicate. Means were compared by non-parametric ANOVA.

TEM analysis revealed that cells transfected with plasmid pA (Figure 2E) and pC (Figure 2F) presented with detectable empty capsid structures but cells transfected with pB did not, perhaps because P12A protein encoded by plasmid pB harboured three additional amino acids after 2A protein self-cleavage between IL-6 and P12A protein, including the amino-terminal proline of 2A protein, upstream of amino-terminal glycine of P1, according to reports [16], [17]. The absorbance values of cells transfected with plasmids pA, pB or pC decreased, but the negative control and irrelevant control absorbance values remained unchanged (Figure 3A). No significant expression level of FMDV capsid protein between pA, pB and pC transfected cells was observed when the cell lysis undiluted.

Characterization of IL-6 production following transfection with either pB or pC produced significantly higher IL-6 levels than untransfected controls, suggesting that transfection of BHK-21 cells with pB and pC resulted in significant levels of IL-6 production (Figure 3B). However, there is no significantly difference for expression level of IL-6 between pB- and pC- transfected cells. In order to confirm the bovine IL-6 reacting well on rodents, we had predicted the reactivity of IL-6 between bovine, rat and human through bioinformatics methods, and concluded that bovine IL-6 may have a well reactivity on rat [18]. However, the reactivity of bovine IL-6 on guinea pig was not predicted because the sequence of guinea pig IL-6 is not available from Genbank.

### Characterization of microparticles properties

**Figure 4:**
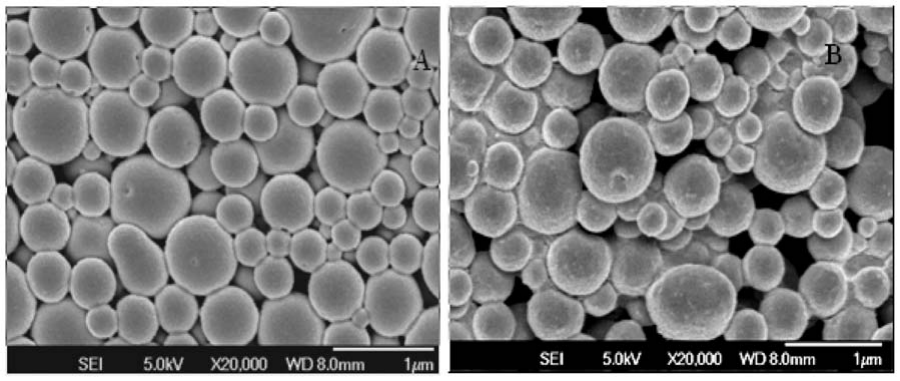
SEM analysis of particle morphology. Particles with plasmids before (a) and after (b) freezing-dry. Magnification 20,000×.

**Figure 5:**
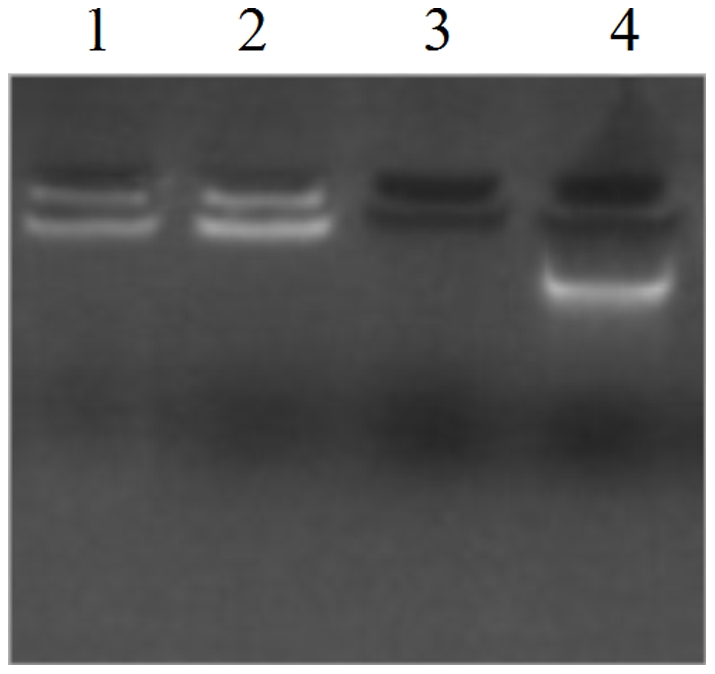
Degradation protection assays of particle-loaded plasmids. Particle-loaded plasmids were incubated with DNaseI. Lane 1, particle loaded plasmid digested with DNase; Lane 2, particle loaded plasmid in the absence of DNaseI digestion; Lane 3, pure plasmid digested with DNaseI; Lane 4, undigested plasmid.

The particle characterization by the scanning electron microscope shows at (Figure 4). The average diameter of the particles determined by ZetaSizer Nano ZS before and after freeze-drying was 462.5 nm and 1975 nm, respectively. The zeta potential of the particles remained unchanged with 41.3 mV at pH 3.0 before and after freeze-drying. The particles protected plasmid DNA from DNase I digestion compared to plasmids that were digested if they were absorbed with the microparticles (Figure 5), suggesting that plasmids absorbed on particles could be effectively delivered to mucosal environments without compromising the integrity of the DNA.

### IL-6 as adjuvant increases the level of serum IgG

**Figure 6:**
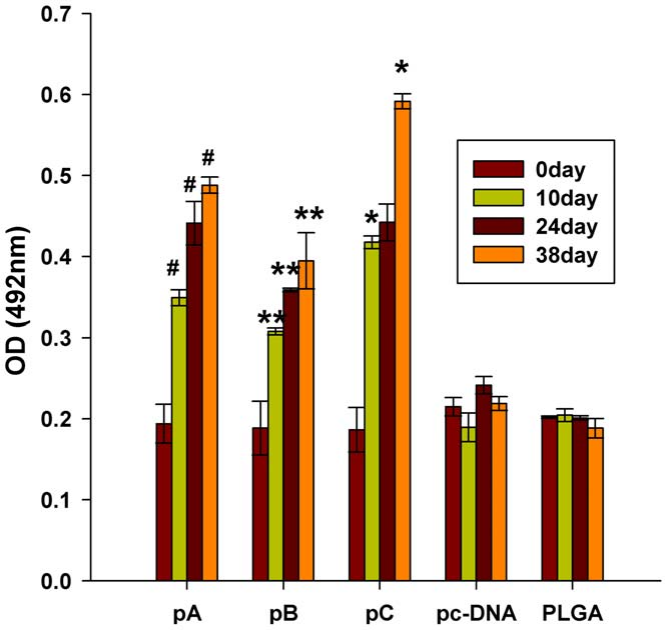
Guinea pig anti-FMDV serum antibody responses. Guinea pigs (n = 5) were immunized with three doses of chitosan PLGA-loaded pA, pB, pC or pc-DNA3.1 at biweekly intervals. Serum samples were collected on days 0, 10, 24 and 38 after primary immunization and the IgG response assessed by ELISA. Data are measured in duplicate and presented as the mean ± SEM. Means were compared by non-parametric ANOVA. Significant results between pA-immunized animals and control groups are indicated by #,Significant results between pC- and pA-immunized animals are indicated by *, and between pB-immunized groups are indicated by **.

Immunized guinea pig serum antibody titers reactive against FMDV strain Asia I were measured by indirect ELISA. Specific anti-FMDV antibody responses were assessed at 0, 10, 24 and 38 days after the first immunization. Interestingly, the highest antibody titers were observed in pC-immunized animals that were statistically higher than titers observed from animals in other groups at day 38 after the first immunization (p < 0.05) (Figure 6).

### IL-6 enhances mucosal immune responses

**Figure 7:**
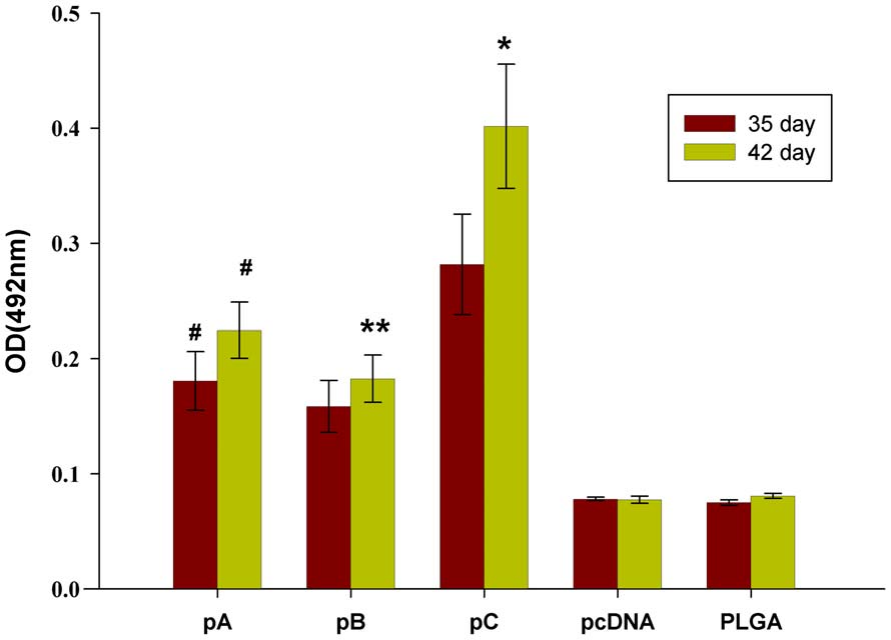
Characterization of FMDV-specific sIgA responses. sIgA FMDV-specific responses were assessed vaginal washes by ELISA. Guinea pigs (n = 5) were vaccinated i.n. with the respective vaccine formulations on days 0, 14 and 28. Vaginal washes were collected on days 35 and 42. Data are measured in duplicate and presented as the mean ± SEM. Means were compared by non-parametric ANOVA. Significant results between pA-immunized animals and control groups are indicated by #, significant results between pC- and pA-immunized animals are indicated by *, and between pB-immunized groups are indicated by **.

On day 35 and 42 days after the first intranasal (i.n.) immunization, sIgA nasal and vaginal responses specific for FMDV were assessed by ELISA. Our results demonstrated the presence of significantly elevated levels of FMDV-specific sIgA antibodies in vaginal wash samples from guinea pigs vaccinated with pC compared to animals vaccinated with pA or pB (Figure 7). The FMDV-specific sIgA antibodies in nasal wash samples shown similar pattern, but there are no significant differences among guinea pigs vaccinated with pC, pA and pB (data not shown).

### sIgA production at mucosal sites

**Figure 8:**
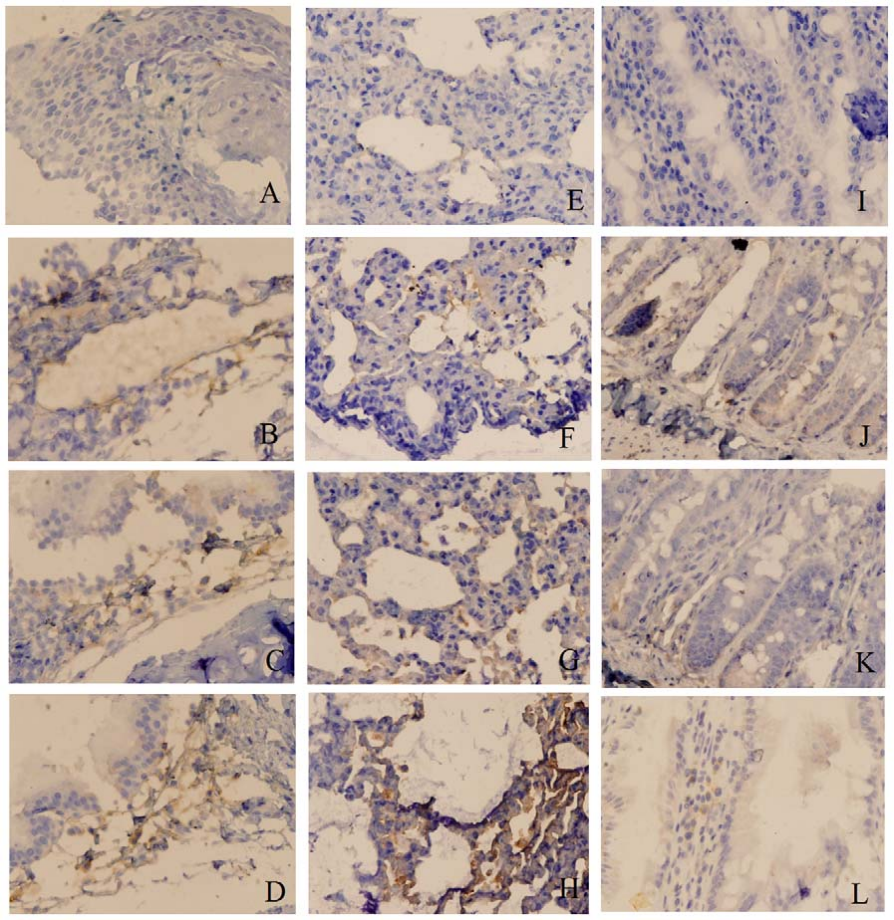
Histologic characterization of sIgA production in tracheas, lungs and small intestines of immunized rats (n = 3). Sections from the respective tissues were obtained from rats that were intranasally vaccinated. Sections were analyzed by immunohistochemistry following incubation with a labeled anti-IgA antibody. Detection of sIgA in respective samples was examined under light microscopy at 40× magnification. Brown staining is representative of sIgA-positive cells from tracheas (A–D), lungs (E–H) and small intestines (I–L). Sections A, E and I were taken from unimmunized animals; sections B, F and J represent tissues harvested from rats immunized intranasally with pA; sections C, G and K represent sections from rats intranasally vaccinated with pB and sections D, H and L represent rats intranasally vaccinated with pC.

Levels of expression and sites of sIgA production in the lungs, tracheas and small intestines of animals in the respective vaccine groups were assessed by immunohistochemical analysis. The highest levels of sIgA expression were observed in all tissues harvested from animals immunized i.n. with pC, followed by animals immunized i.n. with pA and then animals immunized i.n. with pB (Figure 8), paralleling the serum IgA response described above. The significant IgA expression in the alveoli and the intestinal villi supported previous observations [19] suggesting that of the use of DNA encoding IL-6 as a component of the vaccine stimulated the production of higher sIgA concentrations at mucosal sites.

### Characterization of T-cell responses in vitro

**Figure 9:**
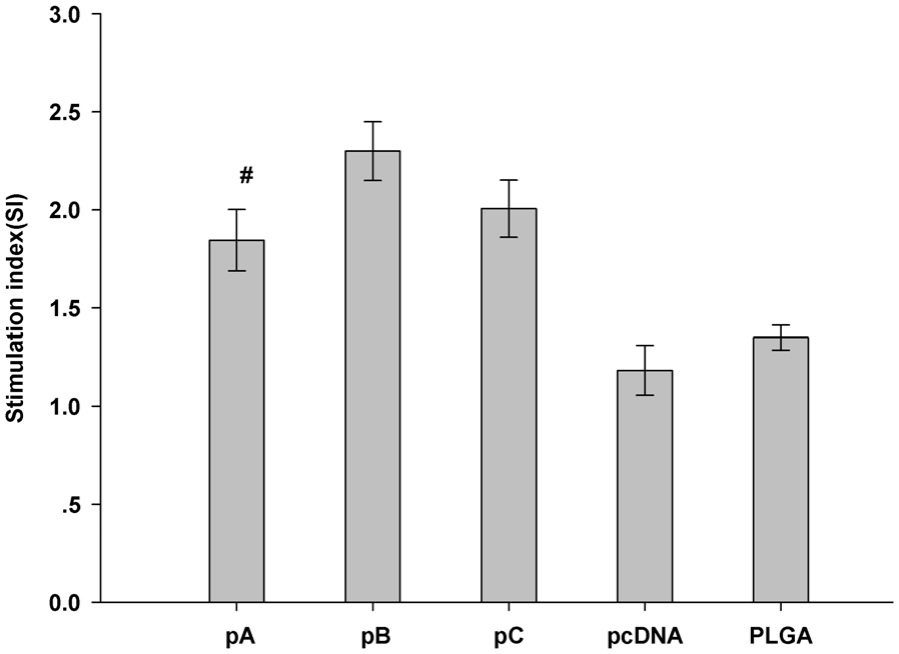
Lymphocyte proliferation. Single lymphocyte suspensions were isolated from rats (n = 6) 7 days after the second immunization, plated in triplicate in a 96-well plate and stimulated *in vitro* for 48 h with inactivated FMDV, Con A (positive control) or with BSA. Means were compared by non-parametric ANOVA. Proliferation was analyzed using the MTT colorimetric assay and proliferation expressed as stimulated index. Significant results between pA-immunized animals and control groups are indicated by #.

Single cell splenocyte suspensions were prepared from rat spleens 7 days after the second immunization. Following stimulation with FMDV, the highest levels of proliferation were observed in splenocytes harvested from pB-immunized animals followed by proliferation of cells harvested from pC- or pA-immunized animals (Figure 9). Proliferation was significantly higher in cells harvested from animals in all the vaccine groups compared to the proliferation observed for cells harvested from the vector control-immunized group.

### Effect of IL-6 on T cell cytokine expression profiles

**Figure 10:**
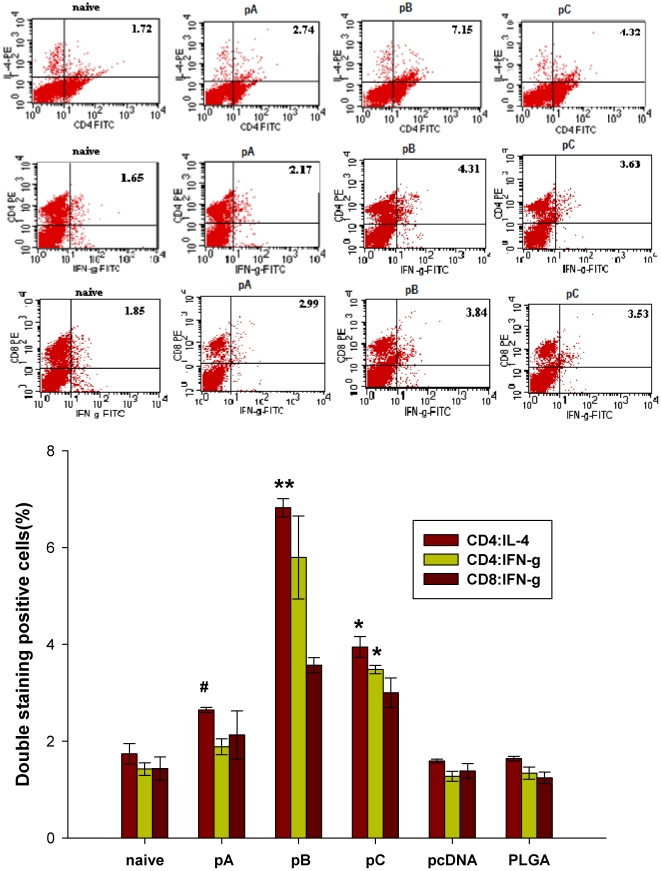
Analysis of cytokine production resulting from antigen-specific stimulation. T cells isolated and purified from the spleens of rats (n = 3) following the second immunization with respective FMDV DNA vaccines were stimulated with inactivated FMDV for 6 h *in vitro* and analyzed by FACS. The percentage of positive cells is shown in each dot-plot in the upper right corner. Data are a representative from three independent experiments. Data are presented as the mean ± SEM. Means were compared by non-parametric ANOVA. Significant results between pA-immunized animals and control groups are indicated by #, significant results between pC- and pA-immunized animals are indicated by *, and between pB-immunized groups are indicated by **.

To understand the effect of IL-6 as a mucosal adjuvant, splenocytes were isolated 7 days after the second immunization and stimulated with inactivated FMDV *in vitro*. Cells were then double-stained with anti-CD4 and anti-IL-4, anti-CD4 and anti-IFN-γ or anti-CD8 and anti-IFN-γ and analyzed in FACS. The highest percentage of antigen-induced IL-4 and IFN-γ producing CD4+ and CD8+ T cells was observed in splenocytes harvested from the pB-immunized groups followed by splenocytes harvested from the pC and pA groups, respectively (Figure 10). In addition, CD4+CD8 +double-positive cells could be induced because CD4+ IFN-γ+ cells or CD8+ IFN-γ+ cells are much more numerous than CD4-IFN-γ+ cells or CD8-IFN-γ+ cells respectively following rats vaccinated with pA, pB, pC compared to control group (Figure 10). CD4+CD8+ double-positive cells induced by vaccination were also reported by [20], [21].

### The effect of IL-6 on DC maturation

**Figure 11:**
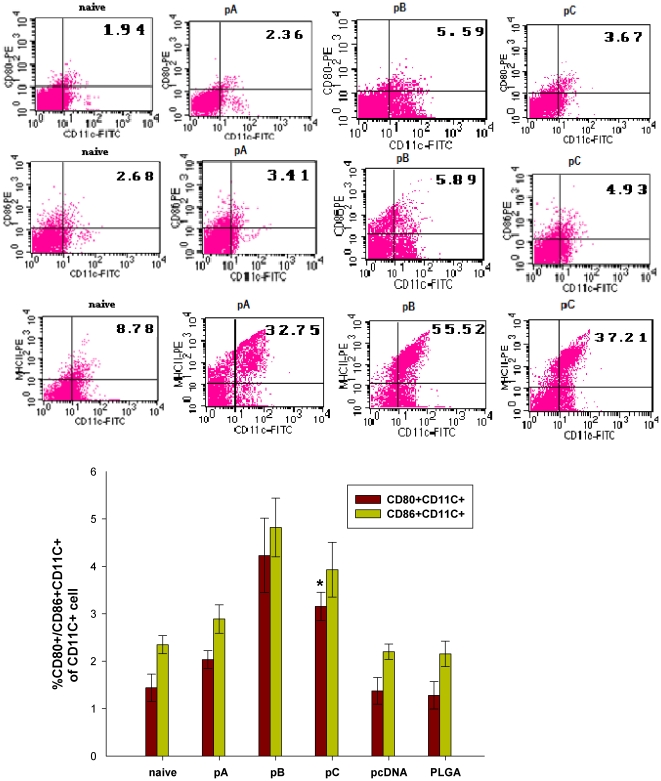
Analysis of DC maturation by FACS. Rat (n = 3) splenocytes were isolated after the second immunization with the respective FMDV DNA vaccine formulations. Harvested cells were double-stained for CD80 and CD11c, CD86 and CD11c. The percentage of double-positive cells in the dot-plot is shown in upper right corner and the gate was set on the CD11c+ cells or events. Data are a representative from three independent experiments. Data are presented as the mean ± SEM. Means were compared by non-parametric ANOVA. Significant results between pA-immunized animals and control groups are indicated by #, significant results between pC- and pA-immunized animals are indicated by *.

Since IL-6 is an excellent candidate for enhancing innate immunity against viral infections [22] and DCs play a critical role in mediating the initiation of cellular immune responses compatible with viral clearance, the role of IL-6 in mediating DC maturation was assessed in the respective vaccine groups. Forty-eight hours post boost immunization, DCs isolated from rats immunized with pB had the highest expression levels of CD80, CD86 and MHC-II followed by expression levels of these markers on DCs harvested from pC- and then pA-immunized rats (Figure 11). DCs expressing MHC-II shown the similar pattern, but there are no significant differences among pA-, pB- and pC- immunized rats (bar graph not shown).

### Characterization of neutralizing viral antibodies

**Figure 12:**
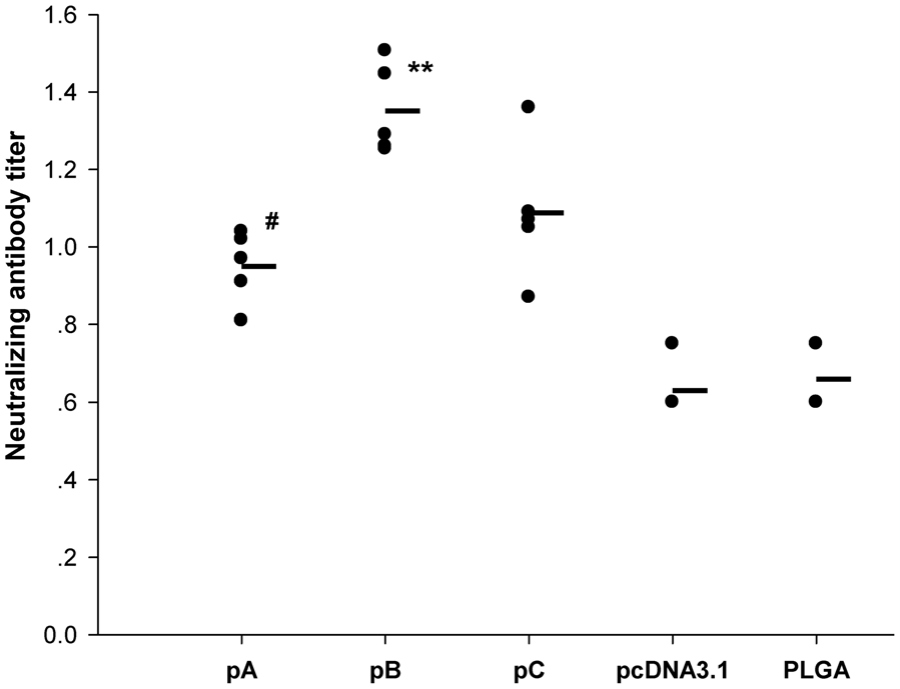
Characterization of FMDV-specific neutralizing antibodies. Serum samples were harvested from guinea pigs (n = 5) on day 38 after the first immunization. Dots represent the level of neutralizing antibodies from each individual animal. Horizontal bars represent the mean neutralizing antibody levels from respective groups. Means were compared by non-parametric ANOVA. Significant results between pA-immunized animals and control groups are indicated by #, significant results between pC- and pB-immunized animals are indicated by **. The results are presented as the mean ± SEM of neutralizing antibody titer.

Since the level of neutralizing antibodies correlates with protection against FMDV infections, the anti-viral neutralizing serum antibody titers from immunized guinea pigs were assessed (Figure 12). The highest levels of neutralizing antibody titers were observed in animals immunized with pB followed by titers in the serum from animals immunized with pC or pA, respectively. Neutralizing titers from animals in all of the vaccine groups were significantly higher than titers observed in serum from animals immunized with vector alone.

### Protection studies

Guinea pigs in all the vaccination groups (n = 5) were subcutaneously challenged with 0.2 ml 100 TCID_50_ of live virus on the left rear foot 2 weeks after the third vaccination. Sixty percent (3/5) of pB-immunized guinea pigs were protected and 20% (1/5) of pC-immnized guinea pigs were protected from FMDV challenge (Table 1). Guinea pigs in the remaining vaccine and control groups developed vesicles in both rear feet 2 days post challenge. The guinea pigs that did not develop secondary vesicles, were judged to be completely protected against challenge with FMDV, suggesting that the pB formulation provided significant levels of protection.

**Table 1:** Protection of guinea pigs (n = 5) against FMDV challenge.

Group^a^		Protection^b^	Prim vesicles^c^	Sec vesicles^c^
A	pc-P12A3C	0/5	5/5	5/5
B	pc-IL2AP12A3C	3/5	5/5	2/5
C	pc-P12AIL3C	1/5	5/5	4/5
D	pcDNA	0/5	5/5	5/5
E	PLGA	0/5	5/5	5/5

Severity of symptoms was based on daily monitoring until 7 days post challenge.

a Guinea pigs were challenged 31 days post vaccination.

b Number of animals without signs of disease/total.

c Number of animals with signs of disease/total.

## Discussion

As reported by [23], [24], utility of nanoparticles for drug delivery do not deposit efficiently in the lungs because of exhalation of a majority of the inhaled dose. Therefore, the nanoparticles- containing microparticles were formulated through freeze-drying the nanoparticles with mannitol added in order to embrace the virtues of both nano- and micro-scale particles, as microparticles turned into nanoparticles rapidly while the mannitol dissolves into water in physiological conditions [25]. This strategy is also regarded as efficient approach to improve the drug absorption by [25], [26].

One question addressed by this study was whether IL-6 is potential as mucosal adjuvant, therefore, pC was constructed with IL-6 located between P12A and 3C sequence in contrast to pA without IL-6 inserted because this strategy is proved to be effective by [27]. Our study showed that pC vaccination increased the levels of antigen-specific sIgA (vaginal wash, p<0.05; nasal washes, p>0.05) that correlated with higher IgA expression levels in the lungs, trachea and small intestines compared to IgA responses observed in pA-immunized rats. These higher sIgA levels elicited following pC vaccination is also consistent with stronger FMDV specific antibody responses(p<0.05) and stronger FMDV neutralizing antibody responses(p>0.05) compared with that following pA vaccination, suggesting that the presence of IL-6 as a vaccine component (acting as a cytokine adjuvant) significantly contributed to the elicitation of mucosal and humoral immune responses. Besides, pC-immunized animals also developed more potent cell-mediated responses, evidenced by the higher percentage of IL-4 (p<0.05) and IFN-γ (p<0.05) producing CD4+ cells and IFN-γ producing CD8+ T cells (p>0.05) and DCs that possessed higher CD80 (p<0.05), CD86 (p>0.05) and MHC II (p>0.05) expression levels compared to pA-immunized rats. In addition, one out of five guinea pigs vaccinated with pC was protected against FMDV challenge, in contrast to none of guinea pigs vaccinated with pA protected. These data suggested that incorporation of IL-6 as part of the FMDV DNA vaccine enhanced mucosal and systemic immune responses specific to FMDV.

Another question addressed by this study was whether targeting FMDV capsid protein to endoplasmic reticulum (ER) can improve the immune responses. Through the signal peptide of IL-6 at the N-terminus, the FMDV capsid protein produced by pB can be targeted to ER but that produced by pC can not according to report [28]. Our results shown that pB vaccination resulted in higher levels of neutralizing antibody titers (p<0.05), a higher percentage of cells responding to antigen stimulation measured by IL-4 (p<0.05) and IFN-γ (p>0.05) production by CD4+ T cells and IFN-γ (p>0.05) production by CD8+ T cells, higher CD80, CD86 and MHC II expression levels from DCs (p>0.05) compared to similar responses from pC-immunized animals. And pB i.n.-immunized animals had the highest protection rate (3/5) compared with (1/5) of pC i.n.-immunized animals. Consistent with our results, animals vaccinated with plasmid DNA encoding VP1 protein of FMDV [29], outer surface protein C of Borrelia burgdorferi [30], HBsAg [31], fragment C of tetanus toxin [32], ovalbumin [33] and gp120 protein of HIV-1 [34] fused to the ER-targeting secretory signal peptide induced stronger immune response compared to that of animals vaccinated with similar antigen without ER-targeting secretory signal peptide fused. Secretion of VP1 [29] and P1 [35] protein of FMDV from cells transfected with plasmid DNA encoding VP1 and P1 fused with ER-targeting secretory signal peptide were reported, therefore, it is tempting to speculate that the FMDV capsid protein could also be secreted from cells transfected with pB but not pC. An attempt to observe whether there are significant differences of FMDV capsid protein levels in culture supernatant of BHK-21 cells transfected with pB and pC was made without success because of severe cellular death (data now shown). It is reported that 3C protein of FMDV could lead to the change in cell morphology closely mimicking FMDV infection leading to cytopathic effect in vitro [36], but there is no report for cellular death in vivo due to 3C protein. Taken together, we speculated the possible mechanism of improving immune responses through ER-targeting strategy could lies in that secreted FMDV capsid proteins are more efficient in presenting for B cells and antigen presenting cells than cytoplasmic FMDV capsid protein.

Interestingly, our results showed that pB vaccination resulted in lower FMDV-specific antibody IgG (p<0.05), IgA (p<0.05) titers (vaginal wash) and obvious lower IgA expression levels in the lungs, trachea and small intestines compared to similar responses from pC-immunized animals, which is inconsistent with neutralizing antibody responses. Similar results have been reported previously. For example, the ratio of neutralizing antibody induced by inactivated FMD vaccine containing 146S antigen at 14 and 28 days post first vaccination is 1.41 and 1.45 respectively, however, the ratio of specific antibody induced by inactivated FMD vaccine and empty capsid-like particles at 14 and 28 days post first vaccination are both much higher than 2 [37]. There is no significant difference between neutralizing antibody induced by inactivated enterovirus 71 antigen and VP1 protein from enterovirus 71, while specific antibody titers induced by inactivated enterovirus 71 are significantly higher than those induced by VP1 [38]. In addition, there are data showing that unmyristoylated FMDV capsid protein can assembly into 17S particles and react with monoclonal antibodies against mature virus [39]. Thus, we speculate that the rapid degradation of un-assembled FMDV capsid protein may lead to insufficient amounts of native protein required to induce a strong FMDV specific antibody response. However, further tests on the efficiency of the 17S capsid protein to induce immune responses compared with 146S or 75S capsid protein should be performed in future.

In summary, IL-6 effectively functioned as a mucosal adjuvant capable of significantly enhancing mucosal and systemic immune responses especially humoral immune responses. Furthermore, pB-immunized animals developed a significantly stronger immune response and provided better protection than animals immunized with the pC formulation, suggesting that differences in FMDV protein (and IL-6) targeting to the ER differed between DNA vaccine formulations. These studies demonstrated that an i.n. immunization strategy comprised of the pB formulation could be developed as an FMDV mucosal vaccine for use in animals following additional testing in protection-challenge experiments and using an animal model that can test the efficacy of these formulations following i.n. challenge.

## Materials and Methods

### Animals

Wistar rats (Lanzhou University, Lanzhou, China) weighing at 200–250 g and guinea pigs (Lanzhou veterinary research institute, China) weighing at 250–300 g were maintained under pathogen-free conditions with free access to pathogen-free food and water. The animal experiments were approved by Gansu Provincial Science and Technology department in China and conducted accordingly. Experiments conformed to the local (Regulations for the administration of affairs concerning experimental animals) and international (Dolan K. 2007 Second Edition of Laboratory Animal Law. Blackwell, UK) guidelines on the ethical use of animals.

### Plasmids

The pcDNA3.1(+) plasmid was purchased from Invitrogen (Carlsbad, CA) and the pMD18-T plasmid purchased from TaKaRa Co. Ltd (Shiga, Japan). Large-scale plasmid preparations were carried out by alkaline lysis using Endofree Qiagen Plasmid-Giga kits (Qiagen, Valencia, CA) according to the manufacturer's instructions.

### Antibodies and fluorescent dye

Fluorescent-conjugated anti-rat monoclonal anti-IL-4-PE, anti-IFN-γ-FITC, anti-CD4-PE, anti-CD4-FITC, anti-CD8-PE and the respective isotype controls were purchased from BD PharMingen (San Diego, CA). Anti-CD11c-FITC, anti-CD80-PE, anti-CD86-PE and anti-MHC-II-PE were purchased from eBioscience (San Diego, CA).

### Construction of the FMDV DNA vaccine

**Figure 13:**
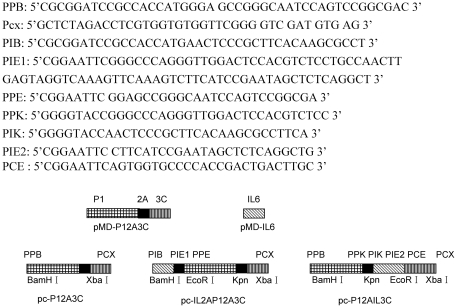
Schematic representation describing the construction of pA, pB and pC.

The FMDV Asia/HeB P12A3C gene fragment was amplified from the pMD-P12A3C plasmid encoding capsid polypeptide (P12A) and 3C protease of FMDV maintained in our laboratory. The bovine IL-6 gene was cloned and inserted into pMD18-T simple vector (TaKaRa Co. Ltd.) as described in Figure 13.

### Transfections

Baby hamster kidney (BHK-21) cells (Boster, Wuhan, China) (4×10^5^) were seeded onto cover slips on six-well plates and incubated at 37°C in a CO_2_ incubator until the cells were 80% confluent. The following day, 10 µg of plasmid DNA in 100 µl of minimal essential media (MEM) with reduced serum (Thermo Fisher, Waltham, MA) was mixed with 6 µl of Lipofectamine™ (Invitrogen). The mixture was then incubated at 20°C for 30 min before adding an additional 800 µl reduced-serum MEM that was then added to the cells. After incubation for 5 h at 37°C in a humidified CO_2_ incubator, 1 ml of medium containing 5% fetal calf serum was added to each well.

Two days after transfections, cells were analyzed for expression of FMDV and IL-6 by the indirect immunofluorescence test (IFAT) and sandwich-ELISA (enzyme-linked immunosorbent assay) [40], [41], [42].

For IFAT, cell monolayers were cultured on coverslips and fixed in cold acetone (−20°C for 30 min). Samples were incubated with rabbit anti-FMDV serum (1∶1000 at 37°C for 30 min) in a humidified chamber and then stained with FITC-conjugated goat anti-rabbit serum (1∶200) for 1 h at 37°C and then observed microscopically.

For sandwich ELISAs designed to detect FMDV protein, cells were washed 48 h after transfection, scraped from the wells and then lysed with lysis buffer (Sangon, Shanghai,China). The lysate was normalized to the total protein content. The lysate was diluted 1∶2 with PBS and added to 96-well flat-bottomed plates (Nunc, Rochester, NY) coated with rabbit anti-FMDV serum overnight at 4°C as above. Subsequently, plates were washed thoroughly with PBST (phosphate buffer saline with 1% tween 20) and anti-FMDV guinea pig sera added to each well. The plates were incubated for 60 min at 37°C and peroxidase-conjugated rabbit anti-guinea pig IgG (Sigma) at a 1∶2000 dilution was added for 1 h at 37°C followed by the addition of OPD-H_2_O_2_. Absorbance was measured at 492 nm 15 minutes later.

Lysed cells normalized to the total protein content were added to 96-well flat-bottom plates and detection of IL-6 by ELISA carried out as described by the bovine IL-6 kit instruction (Thermo Fisher) just expressing the IL-6 titer from transfected cells in optical density (OD).

### Preparation of plasmid DNA (pDNA)-adsorbed CHT-PLGA

Nanoparticles were prepared using the emulsion-diffusion-evaporation technique [43]. Briefly, 200 mg of PLGA (50 kDa) (Shandong institute of medical instruments, China) were dissolved in 10 ml ethyl acetate at room temperature. The organic phase was added to an aqueous stabilizer mixture containing 100 mg of Poly(vinyl alcohol) (PVA) (Sigma)and 30 mg of chitosan (75% to 85% deacetylated, 50–190 kDa) (Sigma) in 10 ml water under stirring. The emulsion was stirred at room temperature for 3 h before homogenizing at 2 000 g (13500 rpm) for 10 min using an Ultra-Turrax T18 homogenizer (Janke and Kunkel GmbH KG, Staufen, Germany). To this emulsion, water was added under stirring, resulting in nano-precipitation. Stirring was continued in a water bath maintained at 40°C for 24 h to remove organic solvents and the solution was then concentrated and resuspended three times to remove residual PVA. CHT-PLGA nanoparticles were stored at 4°C.

pDNA-loaded CHT-PLGA nanoparticles were prepared by mixing the nanoparticles and plasmid (ratio of particles to plasmid was 120∶1(w/w)) at a nanoparticle concentration of 4 mg/ml in MilliQ-water (pH5). Complex formation were performed at room temperature and allowed to stand for 10 h to allow complexes to form. pDNA-loaded CHT-PLGA nanoparticles were stored at 4°C.

The sample made above was prepared for freeze-drying. The nanoparticles and enough mannitol were added to empty, clean borosilicate vials to achieve a 1∶2 mannitol-to-nanoparticles ratio. The appropriate volume of purified particle solution was added to the vial and mixed gently until mannitol was dissolved. The sample was allowed to freeze-dry at −40°C for 48 hours (Hitachi Ltd, Japan), followed by a second drying cycle at a temperature of 20°C for about 8 h. The obtained powder was stored under refrigerated conditions until further use.

### Characterization of nano/microparticle properties

Particle characteristics were determined before and after freeze-drying. Particles were characterized with respect to size and surface charge by measuring the zeta potential using a ZetaSizer Nano ZS (Malvern Instruments Ltd., Worcestershire, UK). Measurements were based on photon correlation spectroscopy at 25°C and a 90 degree scattering angle. All measurements were performed in triplicate at 25°C using the standard settings for water as the dispersion medium.

Nanoparticles with plasmid were deposited on glass, dried for 15 min and then sprinkled with gold powder. The characteristics of the chitosan–plasmid complex surface were then observed using Field Emission-scanning electron microscope (Jeol Ltd., Tokyo, Japan).

Nanoparticle-DNA complexes were prepared by mixing nanoparticles with plasmid at a concentration of 10 mg/ml in MilliQ-water. Complex formation studies were performed at room temperature and allowed to stand for 8 h allowing for complex formation. Nanoparticle-DNA complexes were incubated with 100 units of DNase I at 37°C in 100 µl reaction buffer (40 mM Tris–HCl, pH 7.5, 8 mM MgCl_2_, and 5 mM DTT) for 20 min and stopped by 50 µl stop solution (100 mM EDTA, pH 8.0). The same conditions without nanoparticle-DNA complexes were used in the control experiments. The nanoparticle-DNA complexes were electrophored on agarose gels for 30 min at 5 V/cm. Images were acquired using a Geldoc 2000 gel documentation system (Bio-Rad, Munich, Germany) equipped with a UV transluminator.

### Immunization procedures

Rats were randomly divided into 5 groups (n = 6) and guinea pigs randomly divided into 5 groups (n = 5). Animals were immunized i.n. with either PLGA, pcDNA3.1, pA: pc-P12A3C, pB: pc-IL2AP12A3C or pC: pc-P12AIL3C on days 0, 14 and 28 (Table 2). Female guinea pigs and rats were vaccinated i.n. with 3 or 2 times administrations of vaccine at 200 µg plasmid (36 mg in powder) respectively. Female rats were fully anesthetized by intraperitoneal injection of ketamine before immunization. A MicroSprayer (PennCentury, Philadelphia, PA) was used for i.n. liquid delivery. Animals immunized with chitosan-PLGA only were used as negative controls.

**Table 2:** Immunization groups.

Groups	Animals	Number	Vaccine^a^	Adjuvant
A	Rat	6	pc-P12A3C	Chitosan-PLGA
B	Rat	6	pc-IL2AP12A3C	Chitosan-PLGA
C	Rat	6	pc-P12AIL3C	Chitosan-PLGA
D	Rat	6	pcDNA3.1	Chitosan-PLGA
E	Rat	6	None	Chitosan-PLGA
A	Guinea pig	5	pc-P12A3C	Chitosan-PLGA
B	Guinea pig	5	pc-IL2AP12A3C	Chitosan-PLGA
C	Guinea pig	5	pc-P12AIL3C	Chitosan-PLGA
D	Guinea pig	5	pcDNA3.1	Chitosan-PLGA
E	Guinea pig	5	None	Chitosan-PLGA

a Plasmids were purified and formulated in PBS at 1 µg/µl. Guinea pigs and rats were vaccinated intranasally 3 or 2 times respectively.

### Assessment of humoral responses

ELISAs were used to detect antibody levels against FMDV in sera, vaginal and nasal washes of immunized rats. Vaginal and nasal wash samples were collected by washing the vaginal and nasal cavities of guinea pigs with 100 µl of sterile PBS respectively. Ninety-six-well microtiter plates were coated with inactivated FMDV (100 µl/well) in 0.05 M bicarbonate buffer (pH 9.6) at 4°C overnight. Wells were then blocked with 0.1% of FBS (fetal bovine serum) in 3% BSA (bovine serum albumin)–PBST at 37°C for 1 h, washed and a 1∶50 dilution of guinea pigs serum or a 1∶30 dilution of vaginal or nasal washes were added to respective wells. A secondary antibody, horseradish peroxidase-labeled goat anti-guinea pig IgG (Genetex, San Antonio, TX) or horseradish peroxidase-conjugated goat anti-guinea pig IgA (ICL, Inc., Oregon, OR), were diluted 1∶1000 and added into each well and incubated at 37°C for 1 h. TMB tablets (10 mg) (Sigma) were dissolved in 0.025 M phosphate–citrate buffer and added to each well (100 µl), color development stopped by adding 2 M of H_2_SO_4_ after 15 minutes and absorbance determined at 450 nm using a plate reader (BioTek, Vermont, USA). Antibody reactivity was reported as OD values.

### Immunohistochemical analysis

Trachea, lung and small intestine samples were isolated from immunized rats and fixed using 4% paraformaldehyde, 0.1% glutaraldehyde and 0.2% picric acid in 0.1 M PBS (pH 7.2) at room temperature for 48 h. Serial tissue sections (5 µm thickness) were obtained after the tissues were embedded in paraffin. Antigen detection was performed by heating the sections for 10 min at 120°C in 0.1 M sodium citrate buffer (pH 6.0). Subsequent steps were performed as the instruction of Cell and Tissue Staining Kit-Goat Kit (R&D Systems Inc., Minneapolis, US), and goat anti-rat IgA (Novus Biologicals, Littleton, CO) was used as the first antibody. .Positive cells were visualized under a light microscope at 40×.

### T cell proliferation

Six rats from each immunization group (and controls) were sacrificed and single lymphocyte suspensions were prepared from their spleens 7 days after the second immunization as described previously [42], [43]. Cells were incubated in triplicate in 96-well plates at 5×10^4^ cells/well in RPMI-1640 plus 5% fetal calf serum (FCS) at 37°C in a 5% CO_2_ incubator. The cells were stimulated for 24 h with 10 µg/ml Con A (positive control), 10 µg/ml inactivated FMDV, 5 µg/ml BSA (nonspecific antigen control) or unstimulated (negative control), respectively. T cell proliferation was evaluated using a Cell Titer 96 aqueous non-radioactive cell proliferation assay according to the manufacturer's instruction (Promega, Madison, WI). MTT solution (Promega) was added to each well (20 µl each well). Proliferating cells convert MTT to formazan salt that can be detected after 4 h incubation. Formazan formation correlates with cell growth and can be measured by determining the OD at 595 nm using a plate reader as described above. Data are expressed as stimulation index (SI), calculated as the mean reading of triplicate wells of antigen-stimulated cells divided by the mean reading of triplicate wells from unstimulated (negative control) wells.

### Intracellular cytokine staining

Three rats from each immunization group were sacrificed 7 days after the second immunization. Single-cell splenic suspensions (5×10^5^ cells/200 µl) were stimulated in 96-well plates with inactivated FMDV (5 µg/ml) and anti-CD28 (eBioscience, San Diego, CA) (5 µg/ml) mAb for 12 h at 37°C in 5% CO_2_, followed by the addition of the monensin (BD PharMingen, San Diego, CA) (2 µg/ml) for 4 h and then washed twice with PBS. Cells were blocked with 1 µl of Fcγ mAb (Abcam, Cambridge, UK) (0.5 µg/ml) for 30 min at 4°C and fixed with 4% paraformaldehyde at 4°C for 15 min before permeabilization with 0.1% saponin (Sigma) at 4°C for 10 min. After rinsed once with PBS, the cells were incubated with anti-CD4-FITC and anti-IL-4-PE, or anti-CD8-PE and anti-IFN-γ-FITC, or anti-CD4-PE and anti-IFN-γ-FITC (or with the corresponding isotype controls) for 30 min at 4°C. The fluorescense intensities were measured using a FACS Calibur flow cytometry and the data analyzed using Cell Questpro Software (BD Biosciences, San Jose, CA).

### DC cell surface co-stimulatory molecules staining

Three rats of each group were sacrificed 7 days after the second immunization, single-cell suspensions (1×10^6^ cells/200 µl) from the spleens were blocked with 2 µl of Fcγ mAb (0.5 µg/ml) for 30 min at 4°C. After one PBS wash, cells were used to stain with isotype controls, or double staining with anti-CD11c-FITC and anti-CD80-PE, or anti-CD11c-FITC and anti-CD86-PE, or anti-CD11c-FITC and anti-MHC-II-PE. The fluorescense intensities were measured using a FACS Calibur flow cytometry and the data analyzed using Cell Questpro Software (BD Biosciences, San Jose, CA).

### Detection of specific neutralizing antibodies against FMDV

Serum samples taken from guinea pigs on 38 days after first immunization were analyzed for neutralizing antibody titers using a neutralization assay with monolayers of BHK-21 cells [44]. Doubling dilutions of serum samples were reacted with 100 TCID_50_ of FMDV Asia/HeB at 37°C for 1 h. Cells were then added as indicators of residual infectivity. Endpoint titers were determined after 72 h incubation at 37°C and were calculated as the reciprocal of the final serum dilution that resulted in the neutralization of the virus activity by 50% (ND50). Titers are expressed as the reciprocal of this serum dilution step.

### Animal infections

Thirty-eight days after primary immunization, guinea pigs were challenged subcutaneously and intradermally in one of rear leg with 0.2 ml 100ID_50_ of FMDV (Asia I strain) and examined for protection against FMD over the following 7 days. Guinea pigs that showed FMD-compatible lesions only at the original injection site were judged to be protected, and those that showed any FMD clinical signs in the other three feet were judged to be unprotected [45].

### Statistic analysis

The data were analyzed to express the mean ± standard errors of the mean (SEM). Differences were considered to be statistically significant at p<0.05.
